# High throughput sequencing in mice: a platform comparison identifies a preponderance of cryptic SNPs

**DOI:** 10.1186/1471-2164-10-379

**Published:** 2009-08-17

**Authors:** Nicole AR Walter, Daniel Bottomly, Ted Laderas, Michael A Mooney, Priscila Darakjian, Robert P Searles, Christina A Harrington, Shannon K McWeeney, Robert Hitzemann, Kari J Buck

**Affiliations:** 1 Research and Development Service, Portland VA Medical Center, Portland, OR, USA; 2 Portland Alcohol Research Center, Oregon Health & Science University, Portland, OR, USA; 3 Department of Behavioral Neuroscience, Oregon Health & Science University, Portland, OR, USA; 4 Department of Medical Informatics and Clinical Epidemiology, Oregon Health & Science University, Portland, OR, USA; 5 Oregon Clinical and Translational Research Institute, Oregon Health & Science University, Portland, OR, USA; 6 Knight Cancer Institute, Oregon Health & Science University, Portland, OR, USA; 7 Gene Microarray Shared Resource, Oregon Health & Science University, Portland, OR, USA; 8 Division of Biostatistics in the Department of Public Health and Preventive Medicine, Oregon Health & Science University, Portland, OR, USA

## Abstract

**Background:**

Allelic variation is the cornerstone of genetically determined differences in gene expression, gene product structure, physiology, and behavior. However, allelic variation, particularly cryptic (unknown or not annotated) variation, is problematic for follow up analyses. Polymorphisms result in a high incidence of false positive and false negative results in hybridization based analyses and hinder the identification of the true variation underlying genetically determined differences in physiology and behavior. Given the proliferation of mouse genetic models (e.g., knockout models, selectively bred lines, heterogeneous stocks derived from standard inbred strains and wild mice) and the wealth of gene expression microarray and phenotypic studies using genetic models, the impact of naturally-occurring polymorphisms on these data is critical. With the advent of next-generation, high-throughput sequencing, we are now in a position to determine to what extent polymorphisms are currently cryptic in such models and their impact on downstream analyses.

**Results:**

We sequenced the two most commonly used inbred mouse strains, DBA/2J and C57BL/6J, across a region of chromosome 1 (171.6 – 174.6 megabases) using two next generation high-throughput sequencing platforms: Applied Biosystems (SOLiD) and Illumina (Genome Analyzer). Using the same templates on both platforms, we compared realignments and single nucleotide polymorphism (SNP) detection with an 80 fold average read depth across platforms and samples. While public datasets currently annotate 4,527 SNPs between the two strains in this interval, thorough high-throughput sequencing identified a total of 11,824 SNPs in the interval, including 7,663 new SNPs. Furthermore, we confirmed 40 missense SNPs and discovered 36 new missense SNPs.

**Conclusion:**

Comparisons utilizing even two of the best characterized mouse genetic models, DBA/2J and C57BL/6J, indicate that more than half of naturally-occurring SNPs remain cryptic. The magnitude of this problem is compounded when using more divergent or poorly annotated genetic models. This warrants full genomic sequencing of the mouse strains used as genetic models.

## Background

With the recent completion of the Perlegen/NIEHS mouse resequencing [1], over ten million mouse single nucleotide polymorphisms (SNPs) are now annotated in the public databases, resulting in a dramatic increase in the genome-wide knowledge of variation among 16 of the most widely used mouse strains. Importantly, this is a lower bound estimate because the C57BL/6J (B6) strain, used for the mouse genome reference sequence (NCBI m37, Apr 2007), is the only mouse strain sequenced in its entirety. Given the proliferation of mouse genetic models (e.g., knockout models, selectively bred lines, heterogeneous stocks derived from standard inbred strains and wild mice) and the recent insurgence of gene expression microarray and phenotypic studies using these mouse models, the impact of naturally-occurring polymorphisms on these data is critical. With the advent of next-generation, high-throughput sequencing (HTS), we are now in a position to determine to what extent polymorphisms remain cryptic (either undiscovered or not previously annotated as a SNP in a specific strain comparison) in various mouse models and assess their impact on downstream analyses. As an example, we demonstrate that cryptic SNPs are prevalent, even between two of the most commonly used and well-annotated inbred mouse strains, B6 and DBA/2J (D2).

The onset of next-generation HTS enabled us to obtain full sequence coverage of a region of chromosome 1 in the D2 and B6 mouse strains. There are several platforms for massively parallel DNA sequencing currently on the market [2], and we took this opportunity to directly compare the same dataset on two of the three most widely used platforms: Illumina (Genome Analyzer) and Applied Biosystems (SOLiD). The Genome Analyzer implements a version of cyclic reversible termination chemistry [3], and similarly, the SOLiD platform uses a self-checking ligation chemistry that maps into color space [4]. Both methods generated short reads that were then realigned to a reference sequence.

The present analyses were limited to a region of chromosome 1 from 171.6 – 174.6 megabases (Mb). This interval was selected for four reasons. First, it is representative of the genome in that it spans discrete regions of high and low SNP densities. Second, it is a gene dense region containing 79 protein coding genes, 2 retrotransposed genes, and 6 noncoding RNA genes. Third, it harbors numerous quantitative trait loci (QTLs) affecting a wide variety of physiological and behavioral phenotypes [for summary, see [5]]. And finally, the low incidence of annotated polymorphisms in a SNP-sparse block has hindered high-resolution mapping in this region [6].

We report that comparisons utilizing two well-annotated mouse genetic models, D2 and B6, predict that more than half of naturally-occurring SNPs remain unknown or not annotated. These cryptic SNPs lead to a high incidence of gene expression microarray false-positive and false-negative results and lead to failures in identifying allelically-variant genes that can underlie QTL phenotypic effects.

## Results and discussion

### D2 BAC contig and sequence

**Figure 1:**
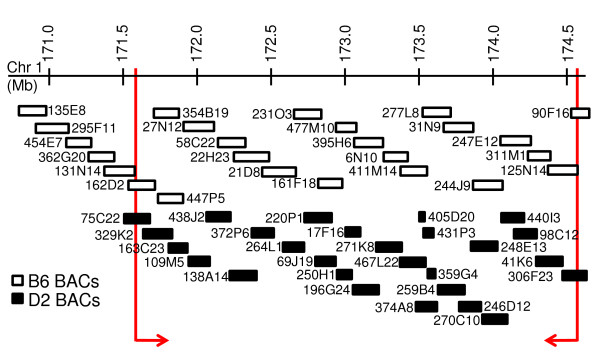
**BAC sequencing coverage**. B6 strain RPCI-23 BACs used for genomic sequencing are denoted as white boxes. D2 strain MM_DBa BACs are indicated as black boxes. Each set of BACs is assembled as an overlapping contig. The 3 Mb (171.6–174.6 Mb) region used for comparisons is bracketed in red.

Using 32 PCR probes spanning the 171.6 – 174.6 Mb interval of chromosome 1, we screened a commercially available D2 strain bacterial artificial chromosome (BAC) library, MM_DBa. End-sequencing of the BACs identified by these PCR probes allowed us to assemble a minimal overlapping contig of 27 D2 BACs (Figure 1). The resulting D2 BAC contig spanned a total of 3.1 Mb from 171,509,721 – 174,625,201 bp on chromosome 1 (Build 37). Contig sequence data from the SOLiD and Genome Anlayzer platforms were assembled via realignment to the Ensembl reference sequence [7] and covered the region surveyed without any large gaps.

### B6 BAC contig and sequence

To evaluate our realignment strategy, we sequenced the corresponding region from the B6 strain, for which full reference sequence is available [7]. We prepared a B6 contig based on public end-sequence data for BAC clones from the RPCI-23 library [8]. The resulting B6 BAC contig spanned 170,806,384 – 174,768,169 bp on chromosome 1 (Figure 1). Using both the SOLiD and Genome Analyzer datasets we attained realignment coverage with the exception of two gaps. The first gap (11 kb) was expected since the RPCI-23 BAC library map lacks annotated coverage in this region. The second gap (100 kb) could be due to an error in the mapping of one or two of the RPCI-23 BACs, since we relied upon reported locations of the B6 BAC library clones, or, alternatively, could be due to one or two clones missing from our pools. These gaps were present in both assemblies indicating a template problem rather than a sequencing discrepancy.

### Comparison of Applied Biosystems and Illumina sequence realignments

Applied Biosytems and Illumina each performed realignments of the D2 and B6 datasets to the public B6 reference sequence [7]. The SOLiD platform produces short reads (35 bp) encoded in a color-space format, and Applied Biosystems used their realignment pipeline specially designed to take advantage of this format for color-space read mapping and downstream analysis including SNP detection. The Genome Analyzer platform produces short read (33 bp) datasets with bases encoded in the standard letter representations, so Illumina carried out a different realignment approach and ran their dataset through the Maq [9] pipeline which includes both realignment and SNP calling procedures. Because both datasets included only short reads, we did not assess potential insertion/deletions.

Comparison of our SOLiD and Genome Analyzer sequence data to the Ensembl reference sequence confirmed that the sequence from both platforms is complete. SOLiD generated 31.8 million reads for the pooled D2 BACs, with 12.4 million (39%) of those mapping to the chromosome 1 target interval. SOLiD generated 32.7 million reads of pooled B6 BACs with 10.5 million (32%) mapping to the chromosome 1 interval. Genome Analyzer generated 14.5 million D2 reads with 9.6 million (66%) mapping to the chromosome 1 interval, and 14.0 million B6 reads with 6.2 million (44%) mapping to the chromosome 1 region. Reads that did not align to the chromosome 1 interval were mostly BAC vector sequence (which was not removed when preparing the DNA), adapters from sequencing chemistry, and bacterial contamination. These are template specific problems and do not reflect upon differences between the sequencing platforms. Additional purification steps would have resulted in fewer unaligned sequences; however, the aligned sequences provided sufficient high read depth coverage, so the exclusion of the unaligned sequences did not adversely affect the present analyses.

**Figure 2:**
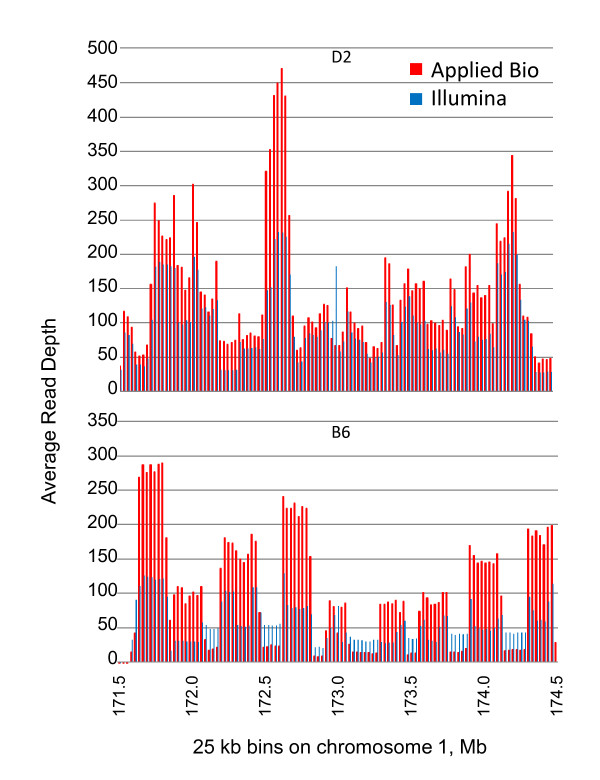
**Read depth for Applied Biosystems' SOLiD (red) and Illumina's Genome Analyzer (blue) realignments**. For D2 and B6 strains for the 171.6–174.6 Mb region of chromosome 1, each tick indicates the average read depth within the corresponding 25,000 bp bin.

Deep sequencing was achieved, with average read depths using the Genome Analyzer of 75 and 51 for D2 and B6, respectively, and 108 and 85, respectively, using SOLiD, for reads aligning to the chromosome 1 interval (Figure 2). Figure 1 illustrates the BAC contig composition. Each BAC was pooled equimolarly and, as expected, we saw higher coverage (i.e., two or more times as many reads) in the areas where two or more BACs overlap because more template was included for those regions (Figure 2). We observed differences in the masking of repetitive sequences in Applied Biosystems vs. Illumina realignments, suggesting that repetitive regions are problematic for both platforms with short read sequencing. Paired-end sequencing was not performed in the present analyses, but could decipher potential ambiguous, repetitive regions in future analyses.

### B6 reference sequence quality

**Figure 3:**
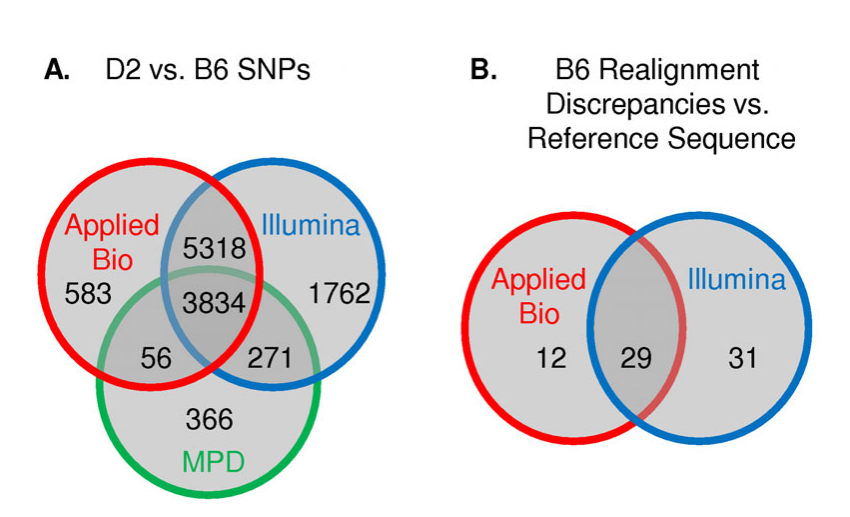
**D2 vs. B6 SNPs and B6 realignment discrepancies vs. reference sequence**. Custom HTS of chromosome 1 (171.6 – 174.6 Mb) data reveals D2 vs. B6 PARC SNPs and realignment discrepancies when compared to the B6 reference sequence. **A) **B6 vs. D2 PARC SNPs identified by Illumina's realignment of Genome Analyzer data (blue circle) and/or Applied Biosystems' realignment of SOLiD data (red circle). D2 vs. B6 SNPs annotated in the Mouse Phenome Database (MPD) are also illustrated for comparison (green circle). Of the 11,824 PARC SNPs found using custom HTS by at least one sequencing method, 7,663 were previously unknown or not annotated as D2. vs. B6 SNPs, demonstrating that public data for D2 vs. B6 SNPs is currently far from complete. **B) **Identification of 12 and 31 provisional discrepancies in the comparison of the B6 reference sequence to Illumina's (blue circle) and Applied Biosystems' (red circle) realignments; and confirmation (i.e., detected by both HTS platforms) of realignment discrepancies at 29 positions when compared to reference sequence.

Although comparison of our custom HTS B6 data to the B6 reference sequence confirmed the high accuracy of the reference sequence (≥ 99.998%), we nonetheless identified a small number of discrepancies in the realignments to the reference sequence. Upon assembly, the Applied Biosystems and Illumina realignments of the B6 sequence reads differed from the B6 reference sequence for 41 and 60 residues (Table 1), respectively; 29 of these differences were consistent in both datasets when compared to the reference sequence (Figure 3). Subsequently, based upon realignment discrepancies called by one or the other platforms, we detected 43 provisional realignment discrepancies between our HTS and the reference sequence. Sequencing on each the SOLiD and Genome Analyzer platforms was completed independently, with assembly algorithms each requiring a minimal depth of 3 reads in order to confirm an allele call. Quality of the nucleotide in question, as well as the quality of the surrounding base calls, was taken into account. Together, these restraints offer high confidence in the B6 allele calls identified by both platforms that differ from the references sequence. Overall, this indicates an exceptionally low discrepancy rate (only 0.001 – 0.002%) between HTS data and the reference sequence and also demonstrates the high quality of the sequence data generated in our analyses. Importantly, our custom HTS sequence data and the B6 reference strain sequence were both from RPCI-23 B6 BACs, which were generated from a pool of five female B6 mice from the Jackson Laboratory. Thus, it is extremely unlikely that strain or template inconsistency contributed to the discrepancies in the realignment of our sequence to the B6 reference sequence.

**Table 1:** Comparison of Illumina and Applied Biosystems realignment data.

	Illumina		SOLiD	
	B6	D2	B6	D2

Total basepairs covered	2898101	3000000	2898101	3000000

Ns in realignment	547	23207	82499	116135

Realignment discrepancies to B6 reference or SNPs detected in D2	128	11221	41	9823

Ambiguous alleles	244	2047	37	635

Results are based upon realignments performed by each vendor for chromosome 1 (171.6–174.6 Mb). N's indicate a 'no call' in the realignment. Realignment discrepancies to B6 reference indicate a mismatch to the reference sequence, and SNP detection in D2 indicates a different allele than the B6 reference sequence. An ambiguous allele indicates that more than one base was called at that base, and since these are inbred animals, those calls are ignored and considered the same as a no call or an 'N'.

### SNP identification and confirmation

In order to compare the SOLiD and Genome Analyzer platforms under optimal conditions for each platform and to compare in a manner the majority of end-users are likely to employ, the manufacturers used parameters determined to be optimal for SNP calling on their particular platform. The Illumina SNP calling method (Maq) relies upon quality scores and applies a filter based on these qualities, read depth, and neighboring SNPs in order to discriminate between a SNP and a base-calling error. Applied Biosystems calls SNPs based on two-base encoding in color-space allowing for more sensitive discrimination between SNPs and base-calling errors. Illumina's method produced fewer no calls (Ns) than Applied Biosystems' method based upon realignments performed by each vendor (Table 1). No nucleotide bias was apparent in the frequency of the calls using the Genome Analyzer and SOLiD platforms. We conclude that because of Maq's probabilistic approach using qualities, more SNPs are called by Illumina/Maq than by Applied Biosystems; however, it remains to be confirmed if Illumina has more false positive calls. It is important to keep in mind that because the vendors used independent mapping and SNP calling approaches, differential results due to the platform cannot be distinguished from those due to the analysis pipeline.

Currently, the Mouse Phenome Database (MPD), which includes dbSNP and Perlegen data among other resources, offers the most inclusive SNP queries for mouse strains, including comparisons of the D2 and B6 strains (see Methods for further explanation of SNP Databases). While, MPD currently annotates 4,527 D2 vs. B6 SNPs in the chromosome 1 interval (171.6–174.6 Mb), custom HTS identified 11,824 SNPs (Figure 3) for the same interval (referred to as PARC SNPs because they were sequenced by one or both platforms in work supported in part by the Portland Alcohol Research Center, PARC). 9,152 (77%) of the 11,824 PARC SNPs identified by custom HTS were identified using both the Applied Biosystems and Illumina realignments (i.e., were identified by two independent experiments) and are therefore of very high quality (Figure 3). 2,033 (17.2%) PARC SNPs were identified only by Illumina's realignment, and 639 (5.4%) PARC SNPs were identified only by Applied Biosystems realignment. Only 271 (13%) of the Illumina-specific PARC SNPs and 56 (10%) of the Applied Biosystems-specific PARC SNPs confirmed known SNPs in MPD.

The SOLiD and Genome Anlayzer datasets were merged for subsequent comparisons. Our results confirmed 4,161 (92%) of the D2 vs. B6 SNPs currently annotated in the MPD public dataset within the chromosome 1 interval, while 236 (5%) of the SNPs reported in MPD for this interval were determined to be false-positive SNPs. The 130 remaining either lie in gaps in our realignments or had ambiguous (undetermined or low quality) calls in the sequence data. Our results identified numerous SNPs not previously annotated in MPD. In fact, our results identified 7,663 new SNPs, more than doubling the number of D2 vs. B6 SNPs found in this chromosome 1 interval.

**Figure 4:**
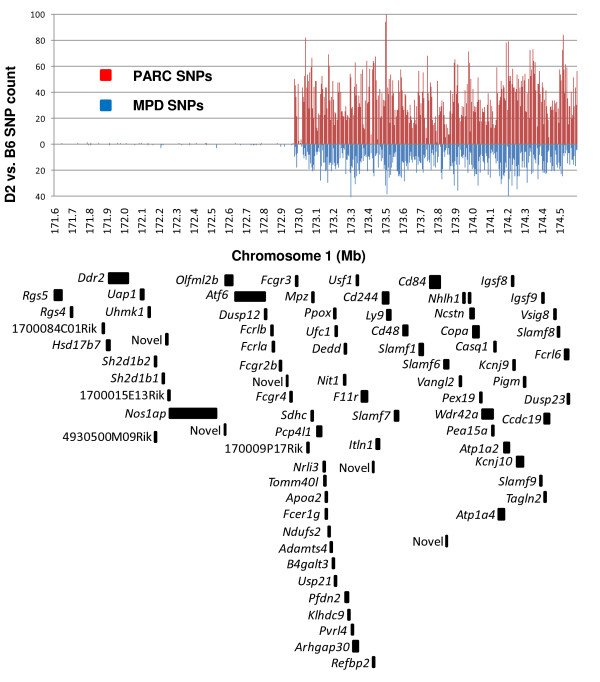
**Density of PARC SNPs and protein coding genes**. B6 vs. D2 PARC SNPs are binned in 5000 bp intervals. The blue lines indicate how many SNPs are currently annotated in the public MPD database, and the red lines show how many PARC SNPs were discovered by custom HTS with realignment of this 3 Mb interval of chromosome 1. SNP dense and SNP sparse regions are apparent. A total of only 16 SNPs were detected between 171.6 – 172.9 Mb, whereas 11,808 SNPs were detected between 172.9 – 174.6 Mb. Below, the black blocks identify the locations of the 79 protein coding genes annotated by Ensembl in this interval.

This chromosome 1 interval spans a clear haplotype break, resulting in a SNP sparse region (171.6–172.9 Mb) and a more distal SNP dense region (172.9 – 174.6 Mb) (Figure 4). The SNP sparse region contains only 16 PARC SNPs based on our custom sequencing, and the SNP dense region harbors 11,808 D2 vs. B6 PARC SNPs. This data further defines the haplotype break between D2 and B6 and, importantly, provides additional genetic markers for fine mapping within the SNP-sparse region which previously was not possible [6]. Additionally, full SNP annotation will inform future SNP array chips allowing for more precise genotyping.

### SNP impact on protein function or expression

We assessed non-synonymous SNPs that result in amino acid changes in proteins because of their clear impact on protein activity. Based on our custom HTS, we found 76 missense (non-synonymous coding) PARC SNPs that changed an amino acid residue between the D2 and B6 strains. 36 of these were new missense SNPs (Table 2). Confirmation experiments (e.g., transcriptome sequencing and PCR directed sequencing) and interrogation of additional datasets [6,10] already have confirmed 30 of the 36 new missense SNPs (Table 2), with the remaining six lacking additional data. In an effort to determine whether the 36 new missense SNPs affect protein function, we used SIFT (Sorting Intolerant From Tolerant) [11], which uses sequence homology to predict whether an amino acid substitution affects protein function, and PolyPhen (Polymorphism Phenotyping) [12], which uses multiple sequence alignments and protein 3D structure to predict protein sequence effects on function. Based on these two prediction methods, eight of the new missense SNPs were predicted to affect protein function, although only one of these amino acid changes was predicted to be damaging by both methods (Table 2). Our data also confirmed 40 missense SNPs annotated in MPD. Finally, five missense SNPs in MPD were not confirmed by our data, including three that had full sequence coverage in our data and were determined to have identical D2 and B6 alleles. Our data indicate that these three are false positive missense SNPs in the public dataset: *Fcgr2b *(K203Q), *Fcgr3 *(A81S), *Itln1 *(Y133F). The other two had ambiguous coverage in our alignments and could not be evaluated: *Fcgr3 *(G72W) and *Itln1 *(N162K).

**Table 2:** Missense B6 vs. D2 PARC SNPs discovered by custom HTS.

**SNP identifier**	**Build 37** **Chromosome 1** **location (bp)**	**Gene**	**Ensembl Protein** **ID(ENSMUS)**	**Amino acid** **change**	**SIFT**	**PolyPhen**
PARCsnp_1.00115^1^	172981853	*Fcgr3*	P00000027964	D244E	tolerated	benign (A)

PARCsnp_1.00877^1^	173088929	*Mpz*	P00000066701	T94A	tolerated	benign (A+S)

PARCsnp_1.01103^1,2^	173126252	*Pcp4l1*	P00000049205	A40T	unknown	benign (A)

PARCsnp_1.01578^1,2,4^	173186807	*Adamts4*	P00000006570	F574Y	tolerated	benign (A)

PARCsnp_1.01735^1^	173214749	*Usp21*	P00000064002	C341R	tolerated	benign (A)

PARCsnp_1.02057^1^	173268767	*Nit1*	P00000106927	L236R*	damaging	probably damaging (A)

PARCsnp_1.02149^1,4^	173290449	*Klhdc9*	P00000056212	E115G*	tolerated	probably damaging (A)

PARCsnp_1.02252^1^	173300263	*Pvrl4*	P00000106917	S12T	unknown	benign (A)

PARCsnp_1.02509^4^	173335292	*Arhgap30*	P00000059389	L458S	tolerated	benign (A)

PARCsnp_1.03202^3,4^	173433896	*Refbp2*	P00000080242	R37S*	tolerated	possibly damaging (A)

PARCsnp_1.03321	173446982	novel	P00000048799	L84V	unknown	benign (A)

PARCsnp_1.14401	173503917	*Cd244*	P00000004829	T56K	tolerated	benign (A+S)

PARCsnp_1.03724^1^	173503965	*Cd244*	P00000004829	D72G	tolerated	benign (A+S)

PARCsnp_1.03725^1^	173504048	*Cd244*	P00000004829	K100Q	tolerated	benign (A+S)

PARCsnp_1.14405^5^	173504085	*Cd244*	P00000004829	T112I*	tolerated	probably damaging (A+S)

PARCsnp_1.03726	173504100	*Cd244*	P00000004829	K117R	tolerated	benign (A+S)

PARCsnp_1.03727^1,5^	173504109	*Cd244*	P00000004829	N120T	tolerated	benign (A+S)

PARCsnp_1.03733^1,4^	173504395	*Cd244*	P00000004829	I186T	tolerated	benign (A)

PARCsnp_1.03736	173504497	*Cd244*	P00000004829	S220L	tolerated	benign (A)

PARCsnp_1.14470	173510893	*Cd244*	P00000004829	S333F*	damaging	benign (A)

PARCsnp_1.03996^1^	173537415	*Ly9*	P00000004827	G14S	tolerated	benign (A)

PARCsnp_1.05632^1,4^	173826546	anon	P00000095074	T98A	unknown	benign (A)

PARCsnp_1.16162^4^	173826619	anon	P00000095074	G122E	unknown	benign (A)

PARCsnp_1.06789^1,2,3,5^	174012752	*Ncstn*	P00000003550	S21F	tolerated	benign (A)

PARCsnp_1.06706^1,2,4^	173996899	*Ncstn*	P00000003550	T678I*	tolerated	possibly damaging (A)

PARCsnp_1.06705^2,4^	173996894	*Ncstn*	P00000003550	V680I	tolerated	benign (A)

PARCsnp_1.06990^1,2,4^	174044893	*Copa*	P00000027833	S761T*	tolerated	possibly damaging (A)

PARCsnp_1.07003^1,2,3,5^	174049099	*Copa*	P00000027833	N984S	tolerated	benign (A)

PARCsnp_1.07779^4^	174185185	*Atp1a4*	P00000007346	I74V	tolerated	benign (A)

PARCsnp_1.07693^1,4^	174174231	*Atp1a4*	P00000007346	N476S	tolerated	benign (A)

PARCsnp_1.07664^1,4^	174170189	*Atp1a4*	P00000007346	M546T	tolerated	benign (A)

PARCsnp_1.08379^1,2,4^	174247711	*Igsf8*	P00000041232	H221R	tolerated	benign (A)

PARCsnp_1.08389^1,2^	174248843	*Igsf8*	P00000041232	T489S	tolerated	benign (A)

PARCsnp_1.08838^1,2,4^	174299836	*Kcnj10*	P00000054356	T262S	tolerated	benign (A)

PARCsnp_1.09831^1,4^	174415000	*Igsf9*	P00000058275	H49R*	tolerated	possibly damaging (A)

PARCsnp_1.10673	174530187	*Fcrl6*	P00000091861	E11D	tolerated	benign (A)

Thirty-six of the seventy-six missense PARC SNPs between the D2 and B6 strains detected by custom HTS were not previously annotated in MPD. Confirmed SNPs are defined as those SNPs detected using two or more independent methods, including the Illumina and Applied Biosystems methods presented here. 30 of the 36 missense SNPs have been confirmed by one or more datasets. ^1^confirmed by both Illumina and Applied Biosystems sequencing pipelines, ^2^confirmed by transcriptome sequencing (see Methods), ^3^confirmed by PCR directed sequencing [6], ^4^confirmed by realignment of Celera raw reads (see SNP Databases in Methods), or ^5^supported by imputed SNPs [24]. Ensembl protein IDs and respective amino acid differences are shown with the B6 amino acid residue indicated first and the D2 residue given last. Each amino acid change was assessed using two programs to determine whether or not the amino acid change is predicted to damage protein function. SIFT uses sequence conservation for predictions and PolyPhen predicts damaging changes using both multiple sequence alignments (A) and/or protein 3D structures (S).

**Figure 5:**
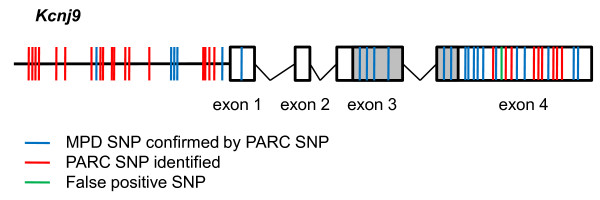
***Kcnj9 *B6 vs. D2 PARC SNPs**. *Kcnj9 *is represented with the coding region shown in the gray boxes and the 5' and 3' untranslated regions (UTRs) in the white boxes. Introns are illustrated as V-lines, and upstream region as a horizontal black line. The blue lines indicate SNPs found in MPD that we confirmed by custom HTS (PARC SNPs). The red lines indicate previously cryptic SNPs, i.e., new PARC SNPs. For *Kcnj9 *(ENSMUST00000062387), we found nine new SNPs in the 3'-UTR, and 18 new SNPs in the 2 kb upstream region, and identified one false positive SNP (green line) in the 3'-UTR. In addition, we confirmed six synonymous coding SNPs, eleven 3'-UTR SNPs, one 5'-UTR SNP, and 5 SNPs within 2 kb upstream of the transcription start site. Additionally, within the introns, we identified 15 new SNPs and confirmed 28 SNPs (not shown).

In addition to the new missense SNPs, we identified 7,627 new PARC SNPs in non-coding regions. These SNPs were not previously known or annotated and may regulate gene and/or protein expression. For example, in the *Kcnj9 *gene, which demonstrates differential transcript expression between the D2 and B6 strains [13], we identified 18 new SNPs within 2 kb upstream of the transcriptional start site as well as nine new SNPs in the 3' untranslated region (Figure 5). We also identified one false-positive SNP annotated in MPD in the 3' untranslated region. Thus, for *Kcnj9 *and other differentially expressed genes, elucidation of cryptic SNPs could identify nucleotide variation that underlies QTL phenotypic effects.

### SNP impact on gene expression microarrays

Naturally occurring genetic polymorphisms dramatically impact hybridization based techniques, including gene expression microarray analyses [14]. With the ability to assess alternative transcript expression, exon microarrays have ten times as many probes as previous gene expression microarrays, so eliminating hybridization bias using SNP masks is increasingly critical. We have developed and applied a complete SNP mask using all of the PARC SNPs found by our custom HTS, which allowed us to rigorously assess differential expression between the D2 and B6 strains. We assessed the impact of 124 SNPs that lie within core probesets on the detection of differential (genotype-dependent) exon expression for genes in the chromosome 1 region of interest using Affymetrix Mouse Exon 1.0 ST array data (for details, please see Mooney et al., companion publication). 629 *core *probesets interrogate this interval. When compared to our unmasked data, masked results were consistent for 141 differentially expressed probesets and 437 non-differentially expressed probesets, but indicated 47 false positive and 4 false negative results due to SNPs.

Furthermore, we overlaid D2 vs. B6 SNPs discovered by our custom HTS with the probe locations of all four types of probesets within the chromosome 1 interval (i.e., core, extended, full, and free) for the Affymetrix Exon 1.0 ST gene expression microarray platform. For chromosome 1 (171.6 – 174.6 Mb), there are 8201 probes and 2126 probesets on the Affymetrix Mouse Exon 1.0 ST array, and we identified 861 probes that spanned at least one SNP encompassing 480 probesets or 23% of the probesets that interrogate this interval (unpublished data). Thus, compared to publicly available D2 vs. B6 SNPs, custom HTS identified 60% more probesets that span SNPs.

## Conclusion

We report that comparisons utilizing even two of the most commonly used mouse genetic models, D2 and B6, predict that more than half of naturally-occurring SNPs remain unknown or not annotated. This is particularly striking given that the present comparison is between the B6 strain, upon which the mouse reference is based [7], and the D2 strain, which is one of the best annotated mouse strains with sequence from Celera and extensive SNP detection data primarily from Perlegen [1]. There are approximately 1.8 million SNPs currently annotated between the D2 and B6 strains in MPD [14]. Thus, cryptic SNPs would have been even more prevalent had we used more divergent or poorly annotated genetic models.

We compare two methods for next-generation HTS. By sequencing the same templates on both Applied Biosystems' SOLiD and Illumina's Genome Analyzer sequencing platforms, we determined that the platforms offer comparable results at a high read depth. More SNPs were called by Illumina/Maq than by Applied Biosystems, but because the vendors used independent mapping and SNP calling approaches, differential results are likely due to differences in the analysis pipelines, as no nucleotide bias in the frequency of the calls made by the Genome Analyzer and SOLiD was apparent.

Mouse models are an invaluable tool for identifying allelic variation that contributes to genetically determined differences in physiology and behavior. However, allelic variation is problematic for follow-up analyses, and removing technical bias resulting from naturally-occurring sequence variation is critical. Previously, we illustrated the impact of SNPs on gene expression microarray analyses [14] and argued that complete SNP masks for gene expression microarray and other hybridization techniques are essential to appropriately interpret these data. Here, we have taken the next step and sequenced a region of two of the most widely used mouse strains in order to determine the comprehensiveness of SNP data. What we have found is that the mouse SNP data currently available is incomplete. In fact, for the D2 vs. B6 strains, we predict that less than half of the true SNPs are currently annotated. As more divergent mouse strains, harboring even more cryptic SNPs, are used in studies, the impact of SNPs on interpreting results will become increasingly problematic. This glimpse at complete sequence data for two strains demonstrates that full genomic sequencing of the mouse strains used as research models is warranted.

## Methods

### D2 BAC library screen

Using 32 chemiluminescent labeled PCR probes designed across a 3 Mb region of chromosome 1 (see Additional File 1 for PCR probe primer pairs), we systematically probed a D2 BAC library consisting of 215,040 clones spotted on 12 nylon filters. The MM_DBa BAC library was generated at Clemson University Genome Institute [15] in 2002 using a single male D2 strain mouse from the Jackson Laboratory where their Genetic Stability Program uses a cryopreservation approach to effectively limit genetic drift and ensure strain stability. 90 BACs were identified in the library screen using the standard Roche DIG chemiluminescent protocol for probing DNA library filters.

### D2 BAC end sequencing

BAC ends were sequenced in order to determine if we had overlap. This was done in several rounds, in which we designed new probes as needed to fill in gaps in our 3 Mb contig. In total, we sequenced the ends of the 99 BAC clones and aligned these BAC end-sequences to the reference mouse sequence. Given that the stringency of the filter hybridizations was variable, that interpretation of positive filter spots was not always straightforward, and that the quality of BAC end-sequencing was not always consistent, we proceeded to confirm each clone we identified. We confirmed a BAC as positive for our region if the end-sequence mapped uniquely to the region of chromosome 1, and the PCR probe mapped in between the end sequences. We confirmed 57 positive BACs for the chromosome 1 region and aligned these to create a minimal contig of 27 overlapping MM_DBa BACs: clones 75C22, 329K2, 163C23, 109M5, 438J2, 138A14, 372P6, 264L1, 69J19, 220P1, 196G24, 250H1, 17F16, 374A8, 271K8, 467L22, 405D20, 431P3, 359G4, 259B4, 246D12, 248E11, 270C10, 440I3, 98C12, 41K6, and 306F23.

### B6 BAC identification

For the B6 BAC contig, we used the RPCI-23 B6 Mouse BAC Library available from Children's Hospital Oakland Research Institute [16], which was generated from a pool of five female B6 strain mice from the Jackson Laboratory, where strain stability is carefully controlled. This is the same library used by the Mouse Genome Sequencing Consortium to generate the B6 reference strain sequence. We used publically available BAC end-sequence for the BAC clones and assembled a minimal contig of 25 overlapping BACs including clones 27N12, 162D2, 90F16, 135E8, 161F18, 244J9, 247E12, 311M1, 354B19, 31N9, 395H6, 231O3, 362G20, 21D8, 295F11, 277L8, 125N14, 447P5, 58C22, 477M10, 22H23, 454E7, 411M14, 131N14, and 6N10.

### DNA preparation

Once the clones were identified, the same DNA preparation protocol was used for the D2 and B6 BAC clones. Vectors were not removed. Each clone was prepared from a glycerol stock by Clemson University using a standard protocol [15]. This resulted in high-quality end-sequence data. We quantified the BAC DNA using a Nanodrop 1000 (Thermo Scientific) and assessed quality of the 260/280 ratios of each BAC clone. For each of two final samples (B6 and D2), BACs were pooled equimolarly and sent to Applied Biosystems (Foster City, CA) and Illumina (San Diego, CA) for next-generation short read DNA sequencing.

### Illumina (Genome Analyzer) sequence and assembly

As per Illumina's requirements, equimolarly pooled BAC DNA was submitted for each B6 and D2 samples: 8.5 ug of B6 BACs and 22.8 ug of D2 BACs. Illumina prepared single read libraries and performed sequencing on the Genome Analyzer I (previously, Solexa) as per standard protocol [17]. Briefly, the sequencing process relies on the amplification of fragmented DNA to form clusters. The sequence content of these clusters is then queried using cycles of fluorescently labeled dNTP addition, detection, and fluorescent groupremoval. The data was optimally assembled by Illumina using Maq (Mapping and Assembly with Quality) [18] with the default parameters in the easyrun script [9]. We used the cns.final.snp output file with minimum read depth of 3 and minimum consensus quality of 30 required to call a SNP for these analyses.

### Applied Biosystems (SOLiD) sequence and assembly

Equimolarly pooled BAC DNA samples were sent to Applied Biosystems as per their requirements: 49.7 ug of B6 BACs and 69.3 ug of D2 BACs. Applied Biosystems prepared two fragment libraries for sequencing from each pool of genomic DNA via standard methods on their SOLiD platform [19]. In short, the SOLiD process attaches clonally-amplified template DNA to a bead and adapter substrate and queries cyclically by adding fluorescently labeled probes specific to two bases, detecting these di-base-calls, removing the fluorescent group, and repeating. This is followed by a primer-reset which shifts the starting primer, allowing different bases to be queried using the above base-calling cycle.SOLiD data was analyzed by Applied Biosystems using their freely available software package. The SOLiD Analysis Tools (SAT) process the array image, perform data filtering, calculate quality values, align to a reference genome, and generate base calls using default parameters [20]. SNPs were called with a minimum read depth of 3 as a requirement.

### Determining non-synonymous SNPs

Non-synonymous SNPs were computed using the Ensembl CDS sequence from UCSC for each transcript. Any SNP falling within coding region and the exon boundaries were used to convert the B6 allele into the D2 allele predicted by the realignments. The coding sequences were then reassembled and translated *in silico *using CLC Sequence Viewer 5 [21]. The amino acid sequences were compared and any differences were noted.

### Transcriptome sequencing

Striatal tissue from D2 (n = 8) and B6 (n = 8) males and females was dissected and total RNA isolated using standard Trizol (Invitrogen) protocol. 3 μg of total RNA was pooled (n = 8) for each strain and sent to Illumina for library construction and standard transcriptome sequencing. The realignment was performed using ELAND with 2 errors allowed in the first 32 bases of a read. Because we had incomplete read coverage for lowly expressed genes, strain-specific sequence results were analyzed for highly expressed genes only. So this dataset offers confirmation of some of the SNPs detected in our DNA sequencing, but did not achieve complete transcriptome coverage.

#### SNP Databases

**1. Mouse Phenome database (MPD)**. The MPD SNP collection [22] contains data for approximately 10 million mouse SNPs for an expansive list of strains from a variety of sources, including datasets such as Broad Institute and Wellcome Trust that are not yet available in other databases. A significant portion of these SNPs are from Perlegen (NIEHS) [1]. Genotype allele tables are generally provided by investigators; however, Perlegen did not include B6 in their analyses. In this case, MPD annotated the reference B6 alleles with the Perlegen data making SNP queries including the B6 strain more inclusive. Specifically, MPD has 4,527 D2 vs. B6 SNPs in the chromosome 1 interval (171.6–174.6 Mb) of interest. All SNPs are mapped to NCBI mouse genome build 37.1 reference assembly (B6). This MPD SNP data build includes annotation from dbSNP 128, Ensembl 48, and NCBI extracted during Dec 2007. The 18 strains with high density SNP data are B6, D2, 129S1/SvImJ, A/J, AKR/J, BALB/cByJ, BTBR_T+_tf/J, C3H/HeJ, CAST/EiJ, FVB/NJ, KK/HlJ, MOLF/EiJ, NOD/ShiLtJ, NZW/LacJ, PWD/PhJ, WSB/EiJ, 129X1/SvJ, and CZECHII/EiJ.

**2. dbSNP Build 128**. dbSNP is maintained at NCBI [23] and includes more than 14 million mouse SNPs. When querying for strain specific polymorphisms, dbSNP identifies 2,903 D2 vs. B6 SNPs in the chromosome 1 interval (171.6–174.6 Mb) of interest. This number is significantly lower than those found in MPD because the Perlegen data does not include B6 alleles, so dbSNP does not retrieve these in the strain specific queries.

**3. Ensembl variation 53**. The Ensembl SNP dataset is queried using Biomart [24] and primarily incorporates dbSNP 128. There are some additional SNPs specific to Ensembl without reference SNP (rs) accession numbers. When querying for strain specific polymorphisms, Ensembl retrieves 2,832 D2 vs. B6 SNPs in the chromosome 1 interval (171.6–174.6 Mb) of interest. Additionally, individual transcript queries in Ensembl include strain variation data that contain realignments of the original strain specific raw reads from Celera (including D2) to the reference B6 sequence. These SNPs provide independent confirmation of some of the SNPs identified by custom HTS and are annotated as confirmation by "Realignment of Celera raw reads" in Table 2.

### Data availability

All of the PARC SNPs discovered from both Applied Biosystems' SOLiD and Illumina's Genome Analyzer sequencing pipelines have been deposited in dbSNP at NCBI under the Handle PARC_SEQ as Computational SNPs (ss#119994841-120015816). D2 BAC end sequence has been submitted to GenBank as GSS (genome survey sequences). Raw sequencing data for SOLiD sequencing has been submitted to GenBank in the SRA (short read archive) without intensity data. Raw sequencing data for the Illumina sequencing is available upon request; however this data was generated before SRA standards were established, and the specific raw files needed for SRF format are no longer available.

## Competing interests

The authors declare that they have no competing interests.

## Authors' contributions

All authors assisted with the concept and design of the study. All of the BAC library screening and sample preparation was conducted by NW, as well as primary manuscript writing. DB analyzed the SOLiD and Illumina sequencing results and assessed the protein coding changes. TL and MM conducted the overlay of the SNPs onto annotated sequences and probes for SNP masks. PD and NW analyzed the SNP lists and masks and deposited data. RS, CH, SKM, RH, and KJB contributed to discussions and manuscript edits. All authors read and approved the final manuscript.

## Supplementary Material

Additional file 1 **D2 BAC probe primer sequences**. Custom PCR primer pairs that were used to generate PCR probes to screen MM_DBa BAC library across chromosome 1 region of interest. All primers are listed 5'-3' orientation. Click here for file
